# Book review: Narrating the Global Financial Crisis: Urban Imaginaries and the Politics of Myth

**DOI:** 10.1177/0042098018796765

**Published:** 2018-09-18

**Authors:** Simon Ferdinand

**Affiliations:** Amsterdam Centre for Cultural Analysis, the Netherlands

Around 2008 (the chronology remains contentious), a series of credit defaults and stock market drops ramified through interconnected financial markets, with consequences that still shape the contemporary socio-political landscape a decade later. In this remarkable new book, cultural and media studies scholar Miriam Meissner underlines how public, political and professional understandings of – and responses to – this crisis have been mediated through pervasive cultural narratives and tropes. *Narrating the Global Financial Crisis* provides a searching, theoretically sophisticated critical account of different discursive constructions of the global financial crisis (which she abbreviates as the ‘GFC’) in media and popular culture. It undertakes close critical analyses of popular narrative films like Oliver Stone’s *Wall Street: Money Never Sleeps* (2010) and David Cronenberg’s (2012) adaptation of Don DeLillio’s novel *Cosmopolis* (2003), television documentaries like Marije Meerman’s *Money and Speed: Inside the Black Box* (2011), novels like John Lancaster’s *Capital* (2012) and a wide range of photojournalism. In doing so, Meissner draws eclectically on theoretical work, ranging from Mikhail Bakhtin’s concept of the chronotrope, to Anthony Vidler’s architectural uncanny, to Jacques Rancière on the politics of dissensus.

The book’s overarching theoretical argument emphasises how financial crisis portrayals constitute a contemporary form of mythology – not in the received sense of being illusory or misleading, but because they crystallise the confusions, contradictions and disjunctions of finance in resonant figurative and narrative forms. Chapter 1 undertakes a formidable survey and synthesis of different conceptual formulations of ‘myth’. From Ernst Crassier, Meissner takes up the notion that myths arise from ‘insecurity, indeterminacy and fear’ (p. 24); from Claude Lévi-Strauss she draws the idea that ‘the world bears irreconcilable contradictions’ (p. 24) and that myth ‘replaces a worldly contradiction with a narrative contradiction that is more bearable’ (p. 25); from Roland Barthes she takes forward a focus on ‘the concrete modalities of its aesthetic composition’, and how mythic figures trigger ‘second order’ cultural connotations beyond their immediate referents (p. 33). Building on these theories, the book assembles a composite conceptualisation of myth ‘as a form of expression that accrues from a state of conflict, or uncertainty, and that, by means of its particular form, conveys a surplus idea – whether critical or not’ (p. 35). Myth ‘does not solve’ or explain the inconsistencies and conflicts that arise from crises like that following 2008, Meissner explains, ‘but rather objectifies, re-articulates and reiterates them narratively’ (p. 20). Myths are a mode of ‘narrative recognition and re-articulation, making contradictions provisionally bearable’ (p. 32). Faced with the contrary fragments of the financial crisis, therefore, media and culture have constructed myths to render it tangible, re-enacting and formalising its central contradictions in ways that partially neuter their frightening power.

Throughout *Narrating the Global Financial Crisis*, Meissner emphasises how representations of the crisis are deeply imbricated with imaginations of the city. Urban imagery, she writes, is ‘strikingly prevalent’ in crisis narratives, providing the settings, actors and objects through which notions of finance are thought and articulated (p. 2). This comes across especially strongly in Chapter 3, which examines the role of architectural scenes and spaces in the ‘late capitalist city’ in crisis representations. Discussion focuses on skyscrapers, whose inscrutable glass facades evoke corporate opacity; skylines, whose jagged silhouettes resemble the plunges and highs of stock indexes; and aerial panoramas of the city, which signal the perceived unaccountability and panoptic power of financial elites. Chapter 4 attends to figures of urban circulation. In popular films, Meissner argues, urban transport systems offer an ‘infrastructural imaginary’ for economic circulation, in which flow signifies success and stasis threatens failure (p. 96). The fleeting proximities and divergent trajectories of commuting, Meissner claims, symbolically articulate the disjointed temporalities of finance capitalism, while sealed vehicles evoke the roving detachment of billionaire traders. The possibility of crashes – vehicular and financial – is constant. These architectural and more generally urban imaginations are drawn from financial crisis portrayals set, primarily, in New York and London. It might be objected, therefore, that the book occludes the involvement of rural areas in interconnected finance economies, and non-Western cities too. However, Meissner explains how these two cities are not only prominent financial centres, but are also established signifiers in the imaginary of global culture and tourism, and thus loom very large in depictions of the financial crisis (p. 13).

Another key emphasis in Meissner’s analysis is the sense of ghostliness or ‘spectral absence’ that pervades mythic depictions of finance, and what this implies for their politics (p. 221). This comes out strongly in Chapter 5, which considers the suburban landscapes of the US subprime mortgage crisis in popular representation. Meissner attends to documentaries showing how an American dream of ‘suburban living and homeownership’, maintained by increasing private indebtedness, disintegrated amid defaults and foreclosures, triggering the wider crisis (p. 139). Meissner’s argument focuses on the peculiar ‘dwelling chronotrope’ (p. 154) – the mode of culturally inhabiting time and space – condensed in representations of heavily financialised ‘unreal estate’ (p. 146). It describes a haunted atmosphere in which ‘material and present’ properties are pervaded, even menaced, by the knowledge that their ‘value and profitability lies in the future, which is highly speculative’ (p. 146). In a context of mortgage defaults, this leads to a stagnated and nostalgic landscape of foreclosed futures, manifested in the ‘new ruins’ of buildings that have never been inhabited (p. 154). Chapter 6, titled ‘Spectres of Finance and the Black Box City’, focuses on explicitly spectral figures in financial crisis narratives, which are rife with suggestions of ‘present, yet incomprehensible, invisible and/or intangible forms of agency’ (p. 186), encompassing ‘black boxes’ of high frequency trading technologies, the ‘invisible hand’ of capitalism, simple market rumours and intimations of a ‘haunted metropolis’ (p. 208).

This is the most critically incisive part of the book, showing clearly how myths of finance underpin different political responses and agendas. Meissner traces the emergence of a ‘post-political discourse of finance’, for which the ontology of financial instruments is so abstract and uncertain, and algorithmic trading so imperceptibly fast, as to totally ‘elude human vision and control’ (p. 186). Finance, here, represents an unfathomable ‘black box’ in which ominous transformations occur, while technical infrastructures of finance assume an autonomous, indeed ‘monstrous agency’ (p. 181). Meissner argues that this ‘post-political’ discourse, by according exaggerated importance to the technical details of financial systems, obscures or downplays the political interests and social structures involved in financialisation. Moreover, she underlines how complexity and incomprehensibility, if heightened in contemporary finance, ‘applies to any form of liberal market totality’ in which unregulated actors interact in large numbers (p. 193). In criticising black box myths, however, *Narrating the Global Financial Crisis* does not aspire to a demystified, objective grasp of finance. Far from it. We come away with a picture in which ‘financialization can only be experienced in a fragmented manner, leaving innumerable “spectral absences” in terms of comprehension and perceptibility’ (p. 221). Meissner’s vision of cultural myths circling untotalisable finance, if appropriately unsettling, allows for both depoliticising and critical forms of myth. Indeed, the book closes by discussing mythic figurations that both perform ‘their own partiality’ (p. 201) and point towards a ‘plural and wide-ranging form of spectral absence’ (p. 225). Instead of fetishising technical obscurity or moralising bankers’ excesses, these myths offer resonant hints and traces of the financial crisis in its unthinkable totality.

*Narrating the Global Financial Crisis* is beautifully bound, formatted and illustrated, in full colour. In providing vivid analyses of diverse cultural representations of finance, informed by cultural and urban theory, it is essential reading for scholars interested in the nexus of economics and culture, and represents an exemplary work of interdisciplinary criticism. As an engagingly written work on a topic of lasting social significance, *Narrating the Global Financial Crisis* is a boon in the seminar room. I have set chapters from the book for two courses, first to exemplify semiotic visual analysis and second to critically explore economic imaginations of globalisation. In both cases the book stimulated wide-ranging discussion. Lastly, to gesture beyond the book’s putative subject matter, Meissner establishes an eminently transferable methodological framework for analysing the cultural forms and political functions of myth. This framework might apply beyond finance, to mythic imaginations of other sources of dissonance, anxiety and conflict in contemporary culture.

